# Reorganization of Brain White Matter in Persistent Idiopathic Tinnitus Patients Without Hearing Loss: Evidence From Baseline Data

**DOI:** 10.3389/fnins.2020.00591

**Published:** 2020-06-16

**Authors:** Qian Chen, Zhaodi Wang, Han Lv, Pengfei Zhao, Zhenghan Yang, Shusheng Gong, Zhenchang Wang

**Affiliations:** ^1^Department of Radiology, Beijing Friendship Hospital, Capital Medical University, Beijing, China; ^2^Department of Otolaryngology Head and Neck Surgery, Beijing Friendship Hospital, Capital Medical University, Beijing, China

**Keywords:** tinnitus without hearing loss, brain white matter, reorganization, tract-based spatial statistics, clinical variables

## Abstract

It remains unknown whether tinnitus or tinnitus-related hearing loss (HL) could indirectly impair or reshape the white matter (WM) of the human brain. We aim to explore the possible brain WM change in tinnitus patients without HL and further to investigate their associations with clinical variables. Structural and diffusion tensor imaging (DTI) of 20 idiopathic tinnitus patients without HL and 22 healthy controls (HCs) were obtained. Voxel-based morphometry (VBM) and tract-based spatial statistics (TBSS) analysis were conducted to investigate the differences in WM volume and integrity between patients and HCs, separately. We extracted WM parameters to determine a sensitive imaging index to differentiate the idiopathic tinnitus patients from the HCs in the early stage. Correlations between the clinical variables and WM indices were also performed in patients. Compared with the controls, the tinnitus patients without HL exhibited significant decreased fractional anisotropy (FA) in the body and genu of corpus callosum (CC), left cingulum (LC) and right cingulum (RC), and right superior longitudinal fasciculus (RSLF) and increase in mean diffusivity (MD) in the body of CC in WM. Moreover, the patients also showed decreases in WM axial diffusivity (AD) in LC, left superior longitudinal fasciculus (LSLF), and right interior cerebellar peduncle (ICP) and increases in radial diffusivity (RD) in the body and genu of CC and RSLF (*p* < 0.05, voxel-level FWE corrected). Furthermore, the increased RD value of the genu of CC is closely associated with the tinnitus handicap inventory (THI) subscale scores. No WMV changes were detected in tinnitus patients. We combined the altered WM integrity index of body and genu of CC and LC and RSLF as an index to differentiate the two groups and reached a sensitivity of 100% and a specificity of 77.3%. Our findings suggest that tinnitus without HL is associated with significant alterations of WM integrity. These changes may be irrespective of the duration and other clinical performance. The combination of diffusion indices of body and genu of CC and LC and RSLF might be used as the potential useful imaging index for the diagnosis of persistent idiopathic tinnitus without HL in the early stage.

## Introduction

Tinnitus, as a perception of sound without an external source, is regarded as a problem of the central nervous system (CNS) (Eggermont and Roberts, 2004). Previous studies have proved that tinnitus can cause significant brain functional and structural changes, which are closely related to the patient’s clinical performance (Ryu et al., 2016; Schmidt et al., 2018; Han et al., 2019). Most of this research has focused on the cortical or resting-state functional alterations (Leaver et al., 2011; Chen et al., 2015a; Liu et al., 2018; Lv et al., 2018), indicating cortical functional changes in some auditory-related areas and altered interactions between multiple auditory-related areas and limbic-related regions in tinnitus. However, studies on brain white matter (WM) changes of the tinnitus are relatively small. Considering the role of the link between the auditory cortex and the limbic system in the generation and maintenance of tinnitus, examining the WM connectivity between these systems may be very important for us to understand the pathophysiology of the disorder.

Currently, there have been a few non-invasive structural studies on brain WM change in tinnitus patients with diverse results (Crippa et al., 2010; Husain et al., 2011; Aldhafeeri et al., 2012; Benson et al., 2014; Seydell-Greenwald et al., 2014; Ryu et al., 2016; Schmidt et al., 2018). Lee et al. (2007) find significantly reduced fractional anisotropy (FA) in superior longitudinal fasciculus in tinnitus patients. Aldhafeeri et al. (2012) also report that tinnitus causes decreased FA in some brain regions or WM fiber bundles, which may be associated with patients’ cognitive deficits, negative emotional suffering, or the imbalance between the two hemispheres in terms of excitation and inhibition. Using tract-based spatial statistics (TBSS) analysis, the study conducted by Seydell-Greenwald et al. (2014) indicates tinnitus-related FA and mean diffusivity (MD) changes in auditory cortical WM, which were correlated with age and hearing loss. Additionally, Ryu et al. (2016) find that the tinnitus group also has higher MD and axial diffusivity (AD) in WM under the auditory cortex and limbic system, and the changed diffusion indices are correlated with patients’ depression. All the studies above prove that tinnitus can result in changes in brain WM integrity, but none of them has made it clear whether the alterations are due to tinnitus or hearing loss (HL).

To date, several studies have analyzed the effect of tinnitus-related HL. For example, Husain et al. (2011) assess WM integrity and find profound changes in the tracts near the auditory cortex in subjects with HL. More specifically, their analyses reveal reduced FA in their HL groups in some auditory or limbic system–related bundles. They speculate these changes could reflect that HL–related sensory deprivation or compensatory mechanisms cause damage to WM tracts or even an expansion of other fibers into these regions. Other research also performs a comparison between groups of subjects with and without tinnitus that are purposely matched with respect to HL (Benson et al., 2014). They also find that the FA elevated in some important hearing or limbic system related tracts and interpret this as the consequence of large-scale changes in excitatory and inhibitory neurotransmission. All of these studies only focus on FA as a measure of tract integrity although, in fact, the interpretation may be problematic due to the high likelihood of crossing fibers and partial-volume effects in the assessed voxels (Seydell-Greenwald et al., 2014).

On the contrary, some research even fails to find any WM changes in tinnitus. An early study investigates the WM connections between the auditory cortex and subcortical nuclei in tinnitus and finds stronger auditory-limbic connectivity in patients, but the employed statistical criteria of their study is lenient as no corrections for multiple comparisons are made (Crippa et al., 2010). Also, Schmidt et al. (2018) even find that there are no differences in diffusion indices in patients when compared with controls, and the severity and duration do not affect FA values as well. They speculate this may be due to the differences in imaging parameters, which do not have the sensitivity to detect the changes.

Based on the above studies, whether tinnitus can cause any change in brain WM and the possible change is due to tinnitus or tinnitus-related HL remains unclear. Therefore, in the present study, we combined used TBSS and VBM to investigate possible changes in brain WM of tinnitus patients without HL and to further explore their relationship with clinical variables. It has been proven that 3–4 years is a kind of turning point in the efficacy of treatment of tinnitus (De Ridder et al., 2007; Plewnia et al., 2007; Langguth et al., 2008). Compared with patients in the early stage of disease (less than 48 months), patients’ brain structure and function with long duration is significantly changed, and this might be the reason why it is difficult for them to be successfully treated (Schlee et al., 2009; Vanneste et al., 2011a,b,c; Liu et al., 2018). Thus, we defined subjects with duration less than 48 months as in the early stage. As prior studies only chose one or two diffusion indices of DTI (Seydell-Greenwald et al., 2014; Ryu et al., 2016; Schmidt et al., 2018), in order to improve the sensitivity of diffusion tensor imaging (DTI) parameters, we apply the four diffusion indices (FA, MD, AD, and radial diffusivity: RD) of DTI to measure the WM integrity of the tinnitus patients.

## Materials and Methods

### Subjects

A total of 20 patients with untreated persistent idiopathic tinnitus were enrolled in this study. (We defined the patients who were diagnosed with idiopathic tinnitus without any treatment as baseline, and after 6 months of sound therapy, as post-treatment.) Twenty-two right-handed healthy volunteers were recruited as healthy controls (HCs). All the patients met the inclusion criteria: persistent idiopathic tinnitus (≥ 6 months persistently and ≤ 48 months) without any history of associated brain diseases confirmed by conventional MRI, no preexisting mental illness or cognitive disorder affecting the structural outcome, and no contradictions to MRI. Tinnitus was present as a single high-/low-pitched sound and two high/low pitched sounds without any rhythm. Based on audiogram results, all the subjects were without hearing loss, which was defined as more than 25 dB hearing loss at frequencies ranging from 250 to 8 kHz (0.250, 0.500, 1, 2, 3, 4, 6, and 8 kHz) in a pure tone audiometry (PTA) examination. We excluded patients with the following criteria: pulsatile tinnitus, hyperacusis on physical examination, otosclerosis, sudden deafness, Ménière’s disease, and other neurological diseases. All the patients were asked to fill in the tinnitus handicap inventory (THI) (Newman et al., 1996) and visual analog scale (VAS) to assess the severity of disease at the time of MRI acquisition. A Beck depression inventory (BDI) was also administered to evaluate the depression at that time. We also evaluated the hearing condition of healthy controls by the audiologists using the questionnaire. Other exclusion criteria for HCs were the same as above.

All the participants were informed of the purpose of the study and gave written consent. The current study was approved by the institutional review board (IRB) of Beijing Friendship Hospital, Capital Medical University (Beijing, China), and was in accordance with the Declaration of Helsinki. The registration number of our study on ClinicalTrials.gov is NCT02774122.

### Image Acquisition

Images were obtained using a 3.0T MRI system (Prisma, Siemens, Erlangen, Germany) with a 64-channel phase-array head coil. The conventional brain axial T2 sequence was acquired prior to the DTI scan to exclude any abnormality in the brain. The DTI experiments were performed using a single-shot, gradient-echo, echo-planar imaging sequence with the following imaging parameters: TR = 8500 ms, TE = 63 ms, matrix = 128 × 128, acquisition voxel size 2 mm × 2 mm × 2 mm, FOV = 224 mm × 224 mm, non-zero *b* value = 1000 s/mm^2^, gradient directions = 64, slice thickness = 2 mm, and bandwidth = 2232 Hz/Px. A total of 74 contiguous slices parallel to the anterior commissure–posterior commissure line were acquired. High-resolution three-dimensional (3-D) structural T1-weighted images were acquired using a 3-D magnetization-prepared rapid gradient-echo sequence (MP-RAGE) with the following parameters: TR = 2530 ms; TE = 2.98 ms; inversion time (TI) = 1100 ms; FA = 7°; number of slices = 192; slice thickness = 1 mm, bandwidth = 240 Hz/Px; FOV = 256 mm × 256 mm; and matrix = 256 × 256; resulting in an isotropic voxel size of 1 mm × 1 mm × 1 mm.

During the scanning process, tight but comfortable foam padding minimized head motion, and earplugs reduced imaging noise. The participants were asked to close their eyes, stay awake, breathe evenly, and try to avoid specific thoughts.

### Image Analysis

#### Data Processing and Diffusion Tensor Imaging (DTI)

Post-processing was performed using TBSS implemented with the FSL 5.0.3 software package (Centre for FMRIB, Oxford University, Oxford, United Kingdom)^1^ (Smith et al., 2006). The following post-processing steps were included: All DTI images were visually checked to eliminate images with apparent artifacts caused by the following: eddy current corrections were applied, and motion artifacts were removed using affine alignment. Next, the non-brain tissues were removed using the brain extract tool (BET); this process reduces the computation times of the DTI fitting and tracking processes and improves the accuracy of the spatial registration. After that, the diffusion tensor of each voxel was fitted using a linear least squares algorithm, and the FA, MD, AD, and RD maps were calculated based on the eigenvalues of diffusion tensors (Raya et al., 2012). For the TBSS analysis, the main procedures were as follows: the entire FA data set was non-linearly coregistered to the Montreal Neurological Institute (MNI) FA template in the FSL database. Next, a mean FA skeleton from the mean FA images of all of the subjects was derived and represented the center of the WM tracts common to the group. An FA threshold of 0.2 was used to involve only the major white matter pathways while eliminating peripheral tracts that are susceptible to misregistration. Finally, each aligned FA map was then projected back onto the skeleton to generate a subject-specific FA skeleton. The processes of non-linear warping and skeleton projection of the FA maps were also applied to MD, AD, and RD maps.

#### Structural Data Processing and Whole Brain Voxel-Based Morphometry Analysis

We did the post-processing of structural data using SPM12. First, all the structural images were checked to make sure no apparent artifacts were caused by factors, such as head motion, susceptibility artifacts, and instrument malfunction. Then, the images were manually reoriented to place the anterior commissure at the origin and the anterior-posterior commissure in the horizontal plane. Next, the structural images were segmented into GM, WM, and cerebrospinal fluid (CSF) areas using the unified standard segmentation option in SPM12. The individual WM and GM components were then normalized into the standard MNI space using the diffeomorphic anatomical registration through exponentiated lie algebra (DARTEL) algorithm (Ashburner, 2007) after segmentation. The normalized WM component was modulated to generate the relative white matter volume (WMV) by multiplication by the non-linear part of the deformation field at the DARTEL step. The resulting WMV images were then smoothed with a 6-mm full-width at half maximum Gaussian kernel.

### Statistical Analyses

For DTI data, TBSS using a non-parametric permutation test (5000 permutations) was performed to compare the DTI indices between the tinnitus patients and the HCs. The permutation test was performed with a fixed-effect general linear model (GLM) with age and gender as nuisance covariates. Statistical significance was set at *p* < 0.05 and corrected for multiple comparisons using the threshold-free cluster enhancement (TFCE) method. For T1 MP-RAGE images, using the GLM in SPM12, a voxel-wise two-sample *t* test was applied to compare the WMV differences in the whole brain between the tinnitus patients and the HCs (voxel-level uncorrected *P* < 0.001, non-stationary cluster-level FWE correction with *P* < 0.05), and age and gender served as nuisance covariates. Next, the regions that exhibited alterations in the DTI indices and WMV in tinnitus patients defined as the regions of interest (ROIs) and the mean values of each ROI of each subject were extracted for subsequent analyses. Finally, partial correlation analysis was performed to explore any potential associations between the patients’ clinical variables (such as the duration, THI scores, BDI scores, and VAS scores) and the indices after removing age and gender effects. Moreover, to test the laterality of tinnitus, we did a one-way ANOVA and *post hoc* analysis among these three different-sided tinnitus groups using the extracted mean values of each ROI that exhibited significant alterations in the DTI indices. The last steps were performed using IBM SPSS Statistics version 23.0 (IBM Inc., Armonk, NY, United States). All data in this study were assessed by Kolmogorov–Smirnov test for normality. Data identified as not normally distributed were analyzed using non-parametric tests.

## Results

### Demographics and Clinical Characteristics

Table 1 provides detailed demographic and clinical data of 20 patients with persistent idiopathic tinnitus and 22 healthy controls. We didn’t find the significant differences of gender and age between the tinnitus and HC groups (both *P*s > 0.01).

**TABLE 1:** Demographic and Clinical Data of 20 Patients with persistent Idiopathic Tinnitus and 22 Healthy Controls.

**Demographic**	**Tinnitus (*n* = 20)**	**Control (*n* = 22)**	***P*-value**
Age, years	39.7 (±12.53)	43.7 (±10.47)	0.437
gender	8 males	10 males	0.509
Tinnitus Handicap Inventory	50.5 (±24.34)	NA	NA
Duration, months	≥6 months & ≤48 months	NA	NA
Type^#^	13: 3: 3: 1	NA	NA
Tinnitus pitch	250 ∼ 8,000 Hz	NA	NA
Laterality	5 right, 6 left, 9 bilateral	NA	NA
Normal hearing	All	All	NA

*NA, not applicable. ^#^1 single high-pitched sound vs. 1 single low-pitched sound vs. 2 high pitched sounds vs. 2 low pitched sounds.*

### Brain WM Changes Between the Tinnitus Patients and HCs and Among Tinnitus Subgroups

**FIGURE 1:**
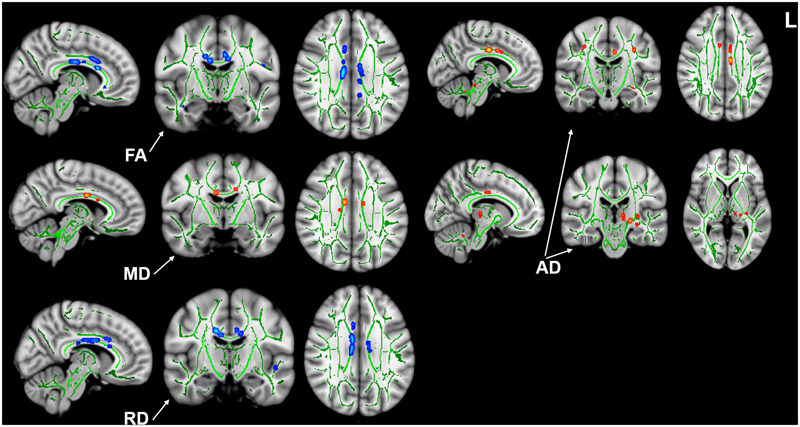
Intergroup differences in white matter integrity between the tinnitus patients and HCs. Patients showed significant decreases of FA in the body and genu of CC, bilateral cingulum, right SLF, and increase of MD in the body of CC. Patients also showed decreases of AD in left cingulum, left SLF, right ICP, and increases of RD in the body and genu of CC and right SLF (*p* < 0.05, voxel-level FWE corrected). HC, healthy control; FA, fractional anisotropy; MD, mean diffusivity; AD, axial diffusivity; RD, radial diffusivity; CC, corpus callosum; SLF, superior longitudinal fasciculus; ICP, interior cerebellar peduncle.

**FIGURE 2:**
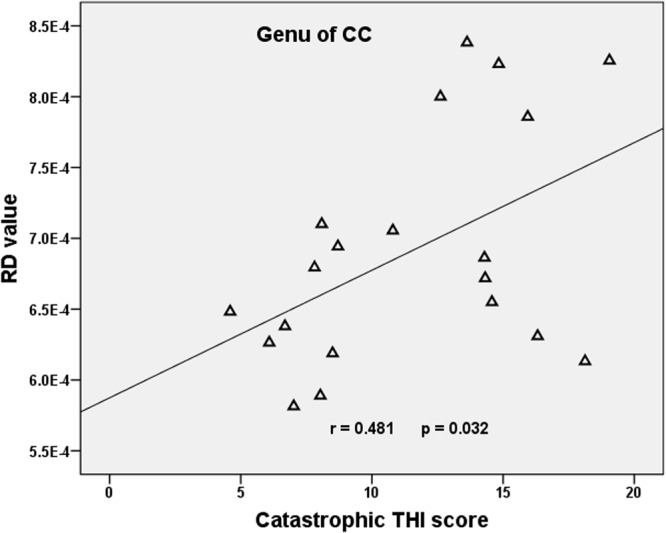
Correlation between diffusion metrics and clinical scale scores in tinnitus patients. Pearson correlation showed positive association between the RD of the genu of CC and the catastrophic THI score (*r* = −0.481, *p* = 0.032; uncorrected). RD, radial diffusivity; CC, corpus callosum; THI, Tinnitus Handicap Inventory.

Compared with the HCs, we found that the tinnitus patients exhibited significant decreases in white matter FA in the body and genu of the corpus callosum (CC), left cingulum (LC) and right cingulum (RC), right superior longitudinal fasciculus (RSLF), and increase in MD in the body of CC. Moreover, the patients also showed decreases in WM AD in LC, left superior longitudinal fasciculus (LSLF), right interior cerebellar peduncle (ICP), and increases in RD in the body and genu of CC and RSLF (voxel-level FWE correction with *p* < 0.05) (Figure 1 and Table 2). No significant differences in WMV were found between the tinnitus and HCs. We also found that there were no significant differences among the three different sided tinnitus subgroups (Supplementary Material). In addition, partial correlation analyses revealed that the RD value of the genu of CC was positively correlated with a subscale of the THI score (*r* = −0.481, *p* = 0.032; uncorrected; Figure 2). No correlations were found between the brain white matter changes and tinnitus duration and other clinical variables.

**TABLE 2:** Regions of significant clusters with changed FA, MD, AD, and RD values in the tinnitus patients compared with the HCs according to the TBSS voxel-based analysis (FWE voxel *p* < 0.05).

**Cluster Index**	**Location of clusters**	**Volume (mm^3^)**	**Mean DTI value**		***p* value**
			**Tinnitus**	**Control**	
FA					
1	Body of CC	141	4.2906	3.3838	0.002
2	Genu of CC	36	1.4034	1.2008	0.011
3	LC	24	0.6656	0.5560	0.003
4	RC	20	0.4924	0.3463	0.002
5	RSLF	11	0.5047	0.4761	0.01
MD					
1	Body of CC	10	0.0024	0.0026	0.022
AD					
1	LC	23	0.0028	0.0026	0.026
2	LSLF	8	0.0012	0.0011	0.005
3	RICP	6	0.0012	0.0011	0.004
RD					
1	Body of CC	60	0.0016	0.0024	0.019
2	Genu of CC	19	0.00069	0.00089	0.014
3	RSLF	10	0.00050	0.00054	0.001

*FA, fractional anisotropy; MD, mean diffusivity; AD, axial diffusivity; RD, radial diffusivity; CC, corpus callosum; LC, left cingulum; RC, right cingulum; RSLF, right superior longitudinal fasciculus; LSLF, left superior longitudinal fasciculus; RICP, right interior cerebellar peduncle; FWE, family wise error; TBSS = tract-based spatial statistics.*

### The Combination of Four Diffusion Parameters of Brain WM Integrity as Diagnostic Index

**FIGURE 3:**
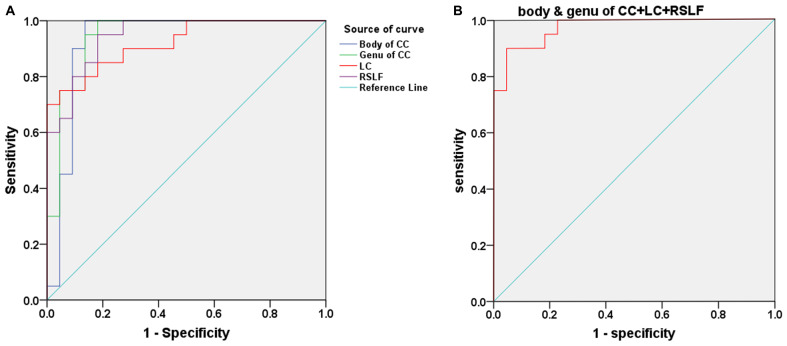
**(A)** Receiver operating characteristic (ROC) curve for uncorrected diffusion metrics in different WM bundles of patients with tinnitus and HCs. An optimal diffusion metric cutoff value of these four bundles was determined at a sensitivity of 100, 100, 90, and 95% and specificity of 86.4, 81.8, 72.7, and 81.8% separately. The area under the curve (AUC) for the ROC were 0.927, 0.943, 0.920, and 0.945 separately (95% confidence intervals from 0.832 to 1, 0.871 to 1, 0.841 to 1, and 0.884 to 1 separately). **(B)** ROC curve for the combination of four diffusion indices in these different WM tracts of patients with tinnitus and HCs. An optimal diffusion metric cutoff value was determined at a sensitivity of 100% and specificity of 77.3%. The AUC for the ROC was 0.973 (95% confidence intervals from 0.934 to 1).

Figure 3A and Table 3 show the sensitivity and specificity of the diffusion indices of the four different white-matter tracts for the tinnitus group and controls. Using the cutoff value of 0.864, 0.818, 0.627, and 0.768, we differentiated the two groups with a sensitivity of 100% and specificity of 86.4% using the body of CC and with a sensitivity of 100% and specificity of 81.8% using the genu of CC and with a sensitivity of 90% and specificity of 72.7% using the LC and with a sensitivity of 95% and specificity of 81.8% using the RSLF separately. The area under the curve (AUC) for the ROC were 0.927 (95% confidence intervals from 0.832 to 1) and 0.943 (95% confidence intervals from 0.871 to 1) and 0.920 (95% confidence intervals from 0.841 to 1) and 0.945 (95% confidence intervals from 0.884 to 1) separately. Figure 3B and Table 3 show the sensitivity and specificity of the combination of four diffusion indices of these white matter tracts for the tinnitus group and controls. Using this cutoff value, 0.773, we differentiated the two groups by a sensitivity of 100% and a specificity of 77.3%. The AUC for the ROC was 0.973 (95% confidence intervals from 0.934 to 1).

**TABLE 3:** Results of receiver operator characteristic (ROC) curve analysis of the combination of four diffusion parameters of brain white matter integrity as a possible diagnostic index.

**Brain white matter integrity**	**AUC**	**Sensitivity**	**Specificity**	***P* value**	**Cutoff**
Body of CC	0.927	100%	86.4%	0.000	0.864
Genu of CC	0.943	100%	81.8%	0.000	0.818
LC	0.920	90%	72.7%	0.000	0.627
RSLF	0.945	95%	81.8%	0.000	0.768
Body & genu of CC + LC + RSLF	0.973	100%	77.3%	0.000	0.773

*ROC, receiver operator characteristic; CC, corpus callosum; LC, left cingulum; RSLF, right superior longitudinal fasciculus; AUC, area under curve.*

## Discussion

By performing TBSS analysis, the present study shows significant increased FA and AD and decreased MD and RD in the distributed WM of the tinnitus patients’ brain; all of these WM tracts are closely related to the information transmission between brain regions or even between hemispheres direct or indirect, especially the auditory information. Moreover, we find that the combination of diffusion indices of several main white-matter tracts could be used to differentiate the persistent tinnitus from controls in the early stage. For the patients, all of them were tested as tinnitus without HL in the classical frequencies in this study. To date, several studies have analyzed HL–related brain reorganization but with inconsistent findings (Crippa et al., 2010; Husain et al., 2011, 2015; Seydell-Greenwald et al., 2014; Schmidt et al., 2018). For example, Husain et al. (2011) assessed the WM integrity and found profound changes in the tracts near the auditory cortex in subjects with HL. More specifically, their analyses revealed reduced FA in their HL groups in some auditory or limbic system–related bundles, which may reflect that HL-related sensory deprivation or compensatory mechanisms caused damage to WM tracts or even an expansion of other fibers into these regions. Other research on tinnitus and tinnitus-related HL found that the FA elevated in some important hearing or limbic system–related tracts and interpreted this as the consequence of large-scale changes in excitatory and inhibitory neurotransmission (Benson et al., 2014). Also, Seydell-Greenwald et al. (2014) found that tinnitus is associated with changes that might reflect increases in auditory and auditory-limbic connectivity while hearing loss is associated with changes in diffusion measures that may reflect decreases in anatomical connectivity of central auditory pathways. Moreover, Schmidt et al. (2018) found no changes in brain white matter between subgroups or compared with HCs. Thus, to investigate the possible tinnitus-caused brain white matter changes and eliminate the effect of hearing loss on the results, all the patients were tinnitus without HL, we believe that the changes in brain white matter integrity may be caused by tinnitus (although we can’t eliminate possible hidden or slight hearing loss in other frequency ranges). Combined with the previous studies, our findings may advance the understanding of the neural pathophysiological mechanisms of tinnitus from the perspective of WM microstructure.

### Brain WM Microstructural Abnormality in Tinnitus Patients

To our knowledge, there are only a few studies that have investigated the brain WM changes in tinnitus so far (Lee et al., 2007; Crippa et al., 2010; Husain et al., 2011; Aldhafeeri et al., 2012; Benson et al., 2014; Seydell-Greenwald et al., 2014; Ryu et al., 2016; Schmidt et al., 2018). Our present study demonstrates a significantly decreased FA and increased RD in the main part (body and genu) of CC WM. For the DTI indices, FA is considered to be the largest in regions in which strongly myelinated fiber tracts run in parallel, permitting free diffusion along but preventing diffusion perpendicular to the fibers. Thus, reductions in myelination or in the number of parallel fibers result in lower FA (Seydell-Greenwald et al., 2014). In addition, RD and MD may also mainly reflect abnormalities in myelination (Bledsoe et al., 2018) although RD may also be affected by axonal loss (Jones et al., 2013) or differences in axonal packing density or diameter (Alexander et al., 2007) although MD to some extent is considered to be inversely related to membrane density and sensitive to cellularity, edema, or necrosis (Alexander et al., 2011). Therefore, we believe that the FA, MD, and RD changes we observed can be the result of demyelination or axonal loss or the differences in axonal packing density caused by tinnitus. More importantly, consistent with prior studies (Aldhafeeri et al., 2012; Seydell-Greenwald et al., 2014), we found changes in WM integrity in the CC. As the largest white matter tract and the major commissure connecting the two hemispheres, it plays a fundamental role in integrating information and mediating complex behaviors (Quigley et al., 2003; van der Knaap and van der Ham, 2011; Hinkley et al., 2012; Roland et al., 2017). The importance of it (especially the anterior part of the CC, such as the genu of it) is embodied in interhemispheric and intrahemispheric communication and the consequent integration of cognitive, emotional, and motor functions. Reduced WM integrity of the CC was reported in some emotional and cognitive disorders such as bipolar disorder (Wang et al., 2008), ADHD (Cao et al., 2010), and PTSD (Jackowski et al., 2008). Moreover, except for the anterior part, the posterior fibers of CC crossing the posterior midbody, isthmus, and splenium transfer somatic sensory, auditory, and visual information (Fabri et al., 2014; Xue et al., 2018), and research even found an enlarged posterior part (the body and splenium) of CC in tinnitus patients, the CC area in which the interhemispheric auditory pathways cross (Diesch, 2012). In addition, Chen et al. (2015b) found stronger interhemispheric connectivity between auditory cortices in tinnitus patients; they speculate that this may be caused by tinnitus and tinnitus-related distress, and the CC played an important role in this process. All of these suggest that tinnitus can not only cause brain structural and functional changes, but also result in changes of brain white matter integrity, and the changed FA and RD of WM in the CC might be caused by the demyelination or axonal loss of persistent tinnitus; then it causes an imbalance between the two hemispheres in terms of simultaneous excitation and inhibition (Aldhafeeri et al., 2012). Also, we found the RD value of the genu of CC positively correlated with the subscale of THI score (catastrophic). A few studies have explored the correlations between WM changes and clinical variables but with controversial results. Several studies found no significant correlations between WM changes and age and clinical performances in tinnitus patients (Lee et al., 2007; Aldhafeeri et al., 2012). However, Ryu et al. (2016) reported that WM integrity in tinnitus patients was associated with depression symptoms, and this finding indicates the WM change in the auditory-limbic circuit attributable to tinnitus and the global influence on WM integrity by tinnitus-related emotional symptoms. As mentioned above, genu is the main part of the anterior CC and the role of it embodied in interhemispheric communication and the consequent integration of cognitive and emotional functions. Thus, this association may indicate the involvement of limbic system dysfunction in tinnitus and also show that the change of white matter may be related to the severity of tinnitus, for which we need to expand the sample in the future for further research on this.

Additionally, a significant decrease in FA and increase in RD was observed in the right superior longitudinal fasciculus (SLF) and an only decreased AD was observed in the left SLF. The SLF is considered to be the major association fiber tract that connects the auditory and frontal cortices, which is consist with three components (I, II, and III) and projects from the frontotemporal and frontoparietal regions in a bidirectional manner, and the inferior longitudinal fasciculus interconnects occipital with ipsilateral temporal lobes (Catani et al., 2002; Makris et al., 2005). The main function of SLF is the integration of information between the speech and auditory regions in the cortex and the integration of auditory and visual association areas with the prefrontal cortex. Therefore, we hypothesized that the changes in SLF may result at least partially in the destruction of the functional connections between some regions in the brain, especially the auditory and prefrontal cortex.

In addition to the three DTI indices (FA, MD, and RD), AD is an important DTI scalar value to measure the WM microstructure as well. As a longitudinal diffusion along axons, AD is indicative of axonal damage and is negatively correlated with axonal injury and positively correlated with axonal repair. Also, studies have proved that AD is usually associated with WM axonal injury in animal models (Song et al., 2003; Alexander et al., 2011) and humans (Smith et al., 2006). In the present study, we found decreased FA and AD in LC and only decreased FA in RC. The cingulum bundle is a prominent WM tract that interconnects frontal, parietal, and medial temporal sites while also linking subcortical nuclei to the cingulate gyrus, which is a major structure in the limbic system and involved in various cognitive functions, including memory, attention, learning, motivation, emotion, and pain perception (Bubb et al., 2018; Jang and Seo, 2018). In addition, DTI reconstructions often suggest a corresponding increase in parietal-temporal connections within the human parahippocampal cingulum bundle (Bubb et al., 2018). Moreover, some structural and functional studies also showed that, as an important aspect of the limbic system, the cingulum (also known as cingulate, including the anterior cingulate and posterior cingulate) plays a key role in several large-scale networks that tinnitus involved in, that is tinnitus emerges as a function of several large-scale networks that bind together various aspects of perception, salience, memory, distress, and audition (De Ridder et al., 2011; Ridder et al., 2011; Schecklmann et al., 2012; Meyer et al., 2016). Therefore, the WM integrity changes in the cingulum suggest that tinnitus is closely related to the limbic system dysfunction.

Together with some previous research (Schlee et al., 2008; Landgrebe et al., 2009; Husain et al., 2011; Leaver et al., 2011; Maudoux et al., 2012; Chen et al., 2015a), all of these findings support the hypotheses that the limbic system plays an important role in the generation of tinnitus, and tinnitus can stress and change neuronal activity and plasticity in the limbic system, which neesd to be further studied in the future. However, no WMV changes found in tinnitus patients, we speculated the reason may be that tinnitus in the early stage may only cause changes in WM integrity, but not have resulted in changes in WMV; further research on this is needed in the future.

### The Combination of Four Diffusion Parameters of Brain WM Integrity as a Diagnostic Index

To date, the pathological changes of WM integrity in the body and genu of CC, the LC, and the SLF have been consistently reported in previous studies but in a separate way (Lee et al., 2007; Aldhafeeri et al., 2012; Benson et al., 2014; Seydell-Greenwald et al., 2014; Ryu et al., 2016). As mentioned above, all these fiber bundles are part of or closely related to the limbic system, and more importantly, they play a role in the onset and development of tinnitus, such as transmitting information between the prefrontal cortices and auditory cortices. Therefore, in the current study, by using the combination of the altered white matter integrity index of body and genu of CC and LC and RSLF as a possible diagnostic index, we could differentiate the two groups at the cutoff value of 0.773 yielding a sensitivity of 100%, specificity of 77.3%, and the AUC value of 0.973. It was an interesting result that could be used as valuable imaging indices for the early diagnosis of persistent idiopathic tinnitus.

### Limitations

Several issues should be noticed in the future. First, the sample size was relatively small, and only right-handed subjects were recruited. Second, as a cross-sectional study, we did not conduct this work as a longitudinal study. Future long-term follow-up studies are necessary to document the full-time course of WM microstructure reorganization in tinnitus. Third, as a pilot study, only tinnitus patients without HL and HCs were recruited in our study. In the future, we will try to design more strict experiments, including tinnitus patients with HL, and that’s exactly what we’re doing recently. Fourth, the definition of tinnitus patients without hearing loss is that there is no hearing loss in the generally recognized frequencies from 250 to 8 kHz; further studies are needed in the future with more accurate instruments to eliminate some possible hidden or slight hearing loss in other frequency ranges.

## Conclusion

In conclusion, we identified that tinnitus without HL can result in significant alterations in brain white matter microstructure, and some of the changes were related to the tinnitus severity. The changes in patients’ WM integrity suggested that there is a close relationship between tinnitus and the limbic system. Moreover, the combination of diffusion parameters of body and genu of CC and LC and RSLF could be used as a valuable imaging index for the diagnosis of early persistent idiopathic tinnitus without HL. These findings added new evidence for the understanding of pathophysiological mechanism of tinnitus.

## Data Availability Statement

The datasets generated for this study are available on request to the corresponding author.

## Ethics Statement

The studies involving human participants were reviewed and approved by the Institutional Review Board (IRB) of Beijing Friendship Hospital, Capital Medical University. The patients/participants provided their written informed consent to participate in this study.

## Author Contributions

QC and ZW were responsible for study conception, acquisition, analysis and interpretation of data, drafting of the manuscript, final approval of the version of the manuscript to be publish, and agreement to be accountable for all aspects of the work. HL, PZ, and ZY were responsible for data analysis, final approval of the version of the manuscript to be publish, and agreement to be accountable for all aspects of the work. SG was responsible for revising the manuscript, final approval of the version of the manuscript to be publish, and agreement to be accountable for all aspects of the work. ZW was responsible for study design, manuscript revision, final approval of the version of the manuscript to be publish, and agreement to be accountable for all aspects of the work. All authors contributed to the article and approved the submitted version.

## Conflict of Interest

The authors declare that the research was conducted in the absence of any commercial or financial relationships that could be construed as a potential conflict of interest.

## Footnotes

1 https://fsl.fmrib.ox.ac.uk/fsl/fslwiki/

## Abbreviations

AD: axial diffusivity
BDI: Beck Depression Inventory
CC: corpus callosum
CSF: cerebrospinal fluid
FA: fractional anisotropy
HC: healthy control
HL: hearing loss
ICP: interior cerebellar peduncle
LC: left cingulum
MD: mean diffusivity
MNI: Montreal Neurological Institute
RC: right cingulum
RD: radial diffusivity
SLF: superior longitudinal fasciculus
THI: Tinnitus Handicap Inventory
WM: white matter
WMV: white matter volume.

## References

[B1] Aldhafeeri F. M. Mackenzie I. Kay T. Alghamdi J. Sluming V. (2012). Neuroanatomical correlates of tinnitus revealed by cortical thickness analysis and diffusion tensor imaging. *Neuroradiology* 54 883–892. 10.1007/s00234-012-1044-6 22614806

[B2] Alexander A. L. Hurley S. A. Samsonov A. A. Adluru N. Hosseinbor A. P. Mossahebi P. (2011). Characterization of cerebral white matter properties using quantitative magnetic resonance imaging stains. *Brain Connect.* 1 423–446. 10.1089/brain.2011.0071 22432902 PMC3360545

[B3] Alexander A. L. Lee J. E. Lazar M. Field A. S. (2007). Diffusion tensor imaging of the brain. *Neurotherapeutics* 4 316–329. 10.1016/j.nurt.2007.05.011 17599699 PMC2041910

[B4] Ashburner J. (2007). A fast diffeomorphic image registration algorithm. *Neuroimage* 38 95–113. 10.1016/j.neuroimage.2007.07.007 17761438

[B5] Benson R. R. Gattu R. Cacace A. T. (2014). Left hemisphere fractional anisotropy increase in noise-induced tinnitus: a diffusion tensor imaging (DTI) study of white matter tracts in the brain. *Hear. Res.* 309 8–16. 10.1016/j.heares.2013.10.005 24212050

[B6] Bledsoe I. O. Stebbins G. T. Merkitch D. Goldman J. G. (2018). White matter abnormalities in the corpus callosum with cognitive impairment in Parkinson disease. *Neurology* 91 e2244–e2255. 10.1212/WNL.0000000000006646 30429273 PMC6329325

[B7] Bubb E. J. Metzler-Baddeley C. Aggleton J. P. (2018). The cingulum bundle: anatomy, function, and dysfunction. *Neurosci. Biobehav. Rev.* 92 104–127. 10.1016/j.neubiorev.2018.05.008 29753752 PMC6090091

[B8] Cao Q. Sun L. Gong G. Lv Y. Cao X. Shuai L. (2010). The macrostructural and microstructural abnormalities of corpus callosum in children with attention deficit/hyperactivity disorder: a combined morphometric and diffusion tensor MRI study. *Brain Res.* 1310 172–180. 10.1016/j.brainres.2009.10.031 19852946

[B9] Catani M. Howard R. J. Pajevic S. Jones D. K. (2002). Virtual in vivo interactive dissection of white matter fasciculi in the human brain. *Neuroimage* 17 77–94. 10.1006/nimg.2002.1136 12482069

[B10] Chen Y. Li X. Liu L. Wang J. Lu C. Yang M. (2015a). Tinnitus and hyperacusis involve hyperactivity and enhanced connectivity in auditory-limbic-arousal-cerebellar network. *eLife* 4:e06576. 10.7554/eLife.06576 25962854 PMC4426664

[B11] Chen Y. Xia W. Feng Y. Li X. Zhang J. (2015b). Altered interhemispheric functional coordination in chronic tinnitus patients. *Biomed. Res. Intern.* 2015 1–8. 10.1155/2015/345647 25789314 PMC4350869

[B12] Crippa A. Lanting C. Dijk P. V. Roerdink J. B. T. M. (2010). A diffusion tensor imaging study on the auditory system and tinnitus. *Open Neuroimaging J.* 4 16–25. 10.2174/1874440001004010016 20922048 PMC2948149

[B13] De Ridder D. De Mulder G. Menovsky T. Sunaert S. Kovacs S. (2007). Electrical stimulation of auditory and somatosensory cortices for treatment of tinnitus and pain. *Prog. Brain Res.* 166 377–388. 10.1016/S0079-6123(07)66036-1 17956802

[B14] De Ridder D. Vanneste S. Congedo M. (2011). The distressed brain: a group blind source separation analysis on tinnitus. *PLoS One* 6:e24273. 10.1371/journal.pone.0024273 21998628 PMC3188549

[B15] Diesch E. (2012). Structural changes of the corpus callosum in tinnitus. *Front. Syst. Neurosci.* 6:17. 10.3389/fnsys.2012.00017 22470322 PMC3312098

[B16] Eggermont J. J. Roberts L. E. (2004). The neuroscience of tinnitus. *Trends Neurosci.* 27 676–682. 10.1016/j.tins.2004.08.010 15474168

[B17] Fabri M. Pierpaoli C. Barbaresi P. Polonara G. (2014). Functional topography of the corpus callosum investigated by DTI and fMRI. *World J. Radiol.* 6 895–906. 10.4329/wjr.v6.i12.895 25550994 PMC4278150

[B18] Han L. Na Z. Chunli L. Yuchen C. Pengfei Z. Hao W. (2019). Baseline functional connectivity features of neural network nodes can predict improvement after sound therapy through adjusted narrow band noise in tinnitus patients. *Front. Neurosci.* 13:614. 10.3389/fnins.2019.00614 31333394 PMC6620714

[B19] Hinkley L. B. Marco E. J. Findlay A. M. Honma S. Jeremy R. J. Strominger Z. (2012). The role of corpus callosum development in functional connectivity and cognitive processing. *PLoS One* 7:e39804. 10.1371/journal.pone.0039804 22870191 PMC3411722

[B20] Husain F. T. Akrofi K. Carpenter-Thompson J. R. Schmidt S. A. (2015). Alterations to the attention system in adults with tinnitus are modality specific. *Brain Res.* 1620 81–97. 10.1016/j.brainres.2015.05.010 25998540

[B21] Husain F. T. Medina R. E. Davis C. W. Szymko-Bennett Y. Simonyan K. Pajor N. M. (2011). Neuroanatomical changes due to hearing loss and chronic tinnitus: a combined VBM and DTI study. *Brain Res.* 1369 74–88. 10.1016/j.brainres.2010.10.095 21047501 PMC3018274

[B22] Jackowski A. P. Douglas-Palumberi H. Jackowski M. Win L. Schultz R. T. Staib L. W. (2008). Corpus callosum in maltreated children with posttraumatic stress disorder: a diffusion tensor imaging study. *Psychiatry Res* 162 256–261. 10.1016/j.pscychresns.2007.08.006 18296031 PMC3771642

[B23] Jang S. H. Seo J. P. (2018). Diffusion tensor tractography studies on injured anterior cingulum recovery mechanisms: a mini-review. *Front. Neurol.* 9:1073. 10.3389/fneur.2018.01073 30581414 PMC6292955

[B24] Jones D. K. Knösche T. R. Turner R. (2013). White matter integrity, fiber count, and other fallacies: the do’s and don’ts of diffusion MRI. *Neuroimage* 73 239–254. 10.1016/j.neuroimage.2012.06.081 22846632

[B25] Landgrebe M. Langguth B. Rosengarth K. Braun S. Koch A. Kleinjung T. (2009). Structural brain changes in tinnitus: grey matter decrease in auditory and non-auditory brain areas. *Neuroimage* 46 213–218. 10.1016/j.neuroimage.2009.01.069 19413945

[B26] Langguth B. Kleinjung T. Frank E. Landgrebe M. Sand P. Dvorakova J. (2008). High-frequency priming stimulation does not enhance the effect of low-frequency rTMS in the treatment of tinnitus. *Exp. Brain Res.* 184 587–591. 10.1007/s00221-007-1228-1 18066684

[B27] Leaver A. M. Renier L. Chevillet M. A. Morgan S. Kim H. J. Rauschecker J. P. (2011). Dysregulation of limbic and auditory networks in tinnitus. *Neuron* 69 33–43. 10.1016/j.neuron.2010.12.002 21220097 PMC3092532

[B28] Lee Y. Bae S. Lee S. Lee J. Lee K. Kim M. (2007). Evaluation of white matter structures in patients with tinnitus using diffusion tensor imaging. *J. Clin. Neurosci.* 14 515–519. 10.1016/j.jocn.2006.10.002 17368031

[B29] Liu Y. Lv H. Zhao P. Liu Z. Chen W. Gong S. (2018). Neuroanatomical alterations in patients with early stage of unilateral pulsatile tinnitus: a voxel-based morphometry study. *Neural Plast.* 2018 1–7. 10.1155/2018/4756471 29681925 PMC5851320

[B30] Lv H. Zhao P. Liu Z. Liu X. Ding H. Liu L. (2018). Lateralization effects on functional connectivity of the auditory network in patients with unilateral pulsatile tinnitus as detected by functional MRI. *Prog. Neuro Psychopharmacol. Biol. Psychiatry* 81 228–235. 10.1016/j.pnpbp.2017.09.020 28941768

[B31] Makris N. Kennedy D. N. McInerney S. Sorensen A. G. Wang R. Caviness V. S. (2005). Segmentation of subcomponents within the superior longitudinal fascicle in humans: a quantitative, in vivo, DT-MRI study. *Cereb. Cortex* 15 854–869. 10.1093/cercor/bhh186 15590909

[B32] Maudoux A. Lefebvre P. Cabay J. E. Demertzi A. Vanhaudenhuyse A. Laureys S. (2012). Connectivity graph analysis of the auditory resting state network in tinnitus. *Brain Res.* 1485 10–21. 10.1016/j.brainres.2012.05.006 22579727

[B33] Meyer M. Neff P. Liem F. Kleinjung T. Weidt S. Langguth B. (2016). Differential tinnitus-related neuroplastic alterations of cortical thickness and surface area. *Hear. Res.* 342 1–12. 10.1016/j.heares.2016.08.016 27671157

[B34] Newman C. W. Jacobson G. P. Spitzer J. B. (1996). Development of the tinnitus handicap inventory. *Archiv. Otolaryngol. Head Neck Surg.* 122:143. 10.1001/archotol.1996.01890140029007 8630207

[B35] Plewnia C. Reimold M. Najib A. Reischl G. Plontke S. K. Gerloff C. (2007). Moderate therapeutic efficacy of positron emission tomography-navigated repetitive transcranial magnetic stimulation for chronic tinnitus: a randomised, controlled pilot study. *J. Neurol. Neurosurg. Psychiatry* 78 152–156. 10.1136/jnnp.2006.095612 16891384 PMC2077659

[B36] Quigley M. Cordes D. Turski P. Moritz C. Haughton V. Seth R. (2003). Role of the corpus callosum in functional connectivity. *Am. J. Neuroradiol.* 24:208. PMC7974118 12591635

[B37] Raya J. G. Horng A. Dietrich O. Krasnokutsky S. Beltran L. S. Storey P. (2012). Articular cartilage: in vivo diffusion-tensor imaging. *Radiology* 262 550–559. 10.1148/radiol.11110821 22106350

[B38] Ridder D. D. Elgoyhen A. B. Romo R. Langguth B. (2011). Phantom percepts: tinnitus and pain as persisting aversive memory networks. *Proc. Natl. Acad. Sci. U.S.A.* 108 8075–8080. 10.1073/pnas.1018466108 21502503 PMC3100980

[B39] Roland J. L. Snyder A. Z. Hacker C. D. Mitra A. Shimony J. S. Limbrick D. D. (2017). On the role of the corpus callosum in interhemispheric functional connectivity in humans. *Proc. Natl. Acad. Sci. U.S.A.* 114 13278–13283. 10.1073/pnas.1707050114 29183973 PMC5740665

[B40] Ryu C. Park M. S. Byun J. Y. Jahng G. Park S. (2016). White matter integrity associated with clinical symptoms in tinnitus patients: a tract-based spatial statistics study. *Eur. Radiol.* 26 2223–2232. 10.1007/s00330-015-4034-3 26449560

[B41] Schecklmann M. Lehner A. Poeppl T. B. Kreuzer P. M. Hajak G. Landgrebe M. (2012). Cluster analysis for identifying sub-types of tinnitus: a positron emission tomography and voxel-based morphometry study. *Brain Res.* 1485 3–9. 10.1016/j.brainres.2012.05.013 22613349

[B42] Schlee W. Hartmann T. Langguth B. Weisz N. (2009). Abnormal resting-state cortical coupling in chronic tinnitus. *BMC Neurosci.* 10:11. 10.1186/1471-2202-10-11 19228390 PMC2649130

[B43] Schlee W. Weisz N. Bertrand O. Hartmann T. Elbert T. (2008). Using auditory steady state responses to outline the functional connectivity in the tinnitus brain. *PLoS One* 3:e3720. 10.1371/journal.pone.0003720 19005566 PMC2579484

[B44] Schmidt S. A. Zimmerman B. Bido Medina R. O. Carpenter-Thompson J. R. Husain F. T. (2018). Changes in gray and white matter in subgroups within the tinnitus population. *Brain Res.* 1679 64–74. 10.1016/j.brainres.2017.11.012 29158175

[B45] Seydell-Greenwald A. Raven E. P. Leaver A. M. Turesky T. K. Rauschecker J. P. (2014). Diffusion imaging of auditory and auditory-limbic connectivity in tinnitus: preliminary evidence and methodological challenges. *Neural Plast.* 2014 1–16. 10.1155/2014/145943 25050181 PMC4090469

[B46] Smith S. M. Jenkinson M. Johansen-Berg H. Rueckert D. Nichols T. E. Mackay C. E. (2006). Tract-based spatial statistics: voxelwise analysis of multi-subject diffusion data. *Neuroimage* 31 1487–1505. 10.1016/j.neuroimage.2006.02.024 16624579

[B47] Song S. K. Sun S. W. Ju W. K. Lin S. J. Cross A. H. Neufeld A. H. (2003). Diffusion tensor imaging detects and differentiates axon and myelin degeneration in mouse optic nerve after retinal ischemia. *Neuroimage* 20 1714–1722. 10.1016/j.neuroimage.2003.07.005 14642481

[B48] van der Knaap L. J. van der Ham I. J. M. (2011). How does the corpus callosum mediate interhemispheric transfer? a review. *Behav. Brain Res.* 223 211–221. 10.1016/j.bbr.2011.04.018 21530590

[B49] Vanneste S. Heyning P. V. Ridder D. D. (2011a). Contralateral parahippocampal gamma-band activity determines noise-like tinnitus laterality: a region of interest analysis. *Neuroscience* 199 481–490. 10.1016/j.neuroscience.2011.07.067 21920411

[B50] Vanneste S. Plazier M. van der Loo E. Van de Heyning P. De Ridder D. (2011b). The difference between uni- and bilateral auditory phantom percept. *Clin. Neurophysiol.* 122 578–587. 10.1016/j.clinph.2010.07.022 20801079

[B51] Vanneste S. van de Heyning P. De Ridder D. (2011c). The neural network of phantom sound changes over time: a comparison between recent-onset and chronic tinnitus patients. *Eur. J. Neurosci.* 34 718–731. 10.1111/j.1460-9568.2011.07793.x 21848924

[B52] Wang F. Kalmar J. H. Edmiston E. Chepenik L. G. Bhagwagar Z. Spencer L. (2008). Abnormal corpus callosum integrity in bipolar disorder: a diffusion tensor imaging study. *Biol. Psychiatry* 64 730–733. 10.1016/j.biopsych.2008.06.001 18620337 PMC2586998

[B53] Xue C. Shi L. Hui S. C. N. Wang D. Lam T. P. Ip C. B. (2018). Altered white matter microstructure in the corpus callosum and its cerebral interhemispheric tracts in adolescent idiopathic scoliosis: diffusion tensor imaging analysis. *Am. J. Neuroradiol.* 39 1177–1184. 10.3174/ajnr.A5634 29674416 PMC7410631

