# Supplementary figures and images for: Reorganization of Brain White Matter in Persistent Idiopathic Tinnitus Patients Without Hearing Loss: Evidence From Baseline Data
Source: Front Neurosci. 2020 Jun 16;14:591. doi: 10.3389/fnins.2020.00591 (PMC7308730; doi:10.3389/fnins.2020.00591)

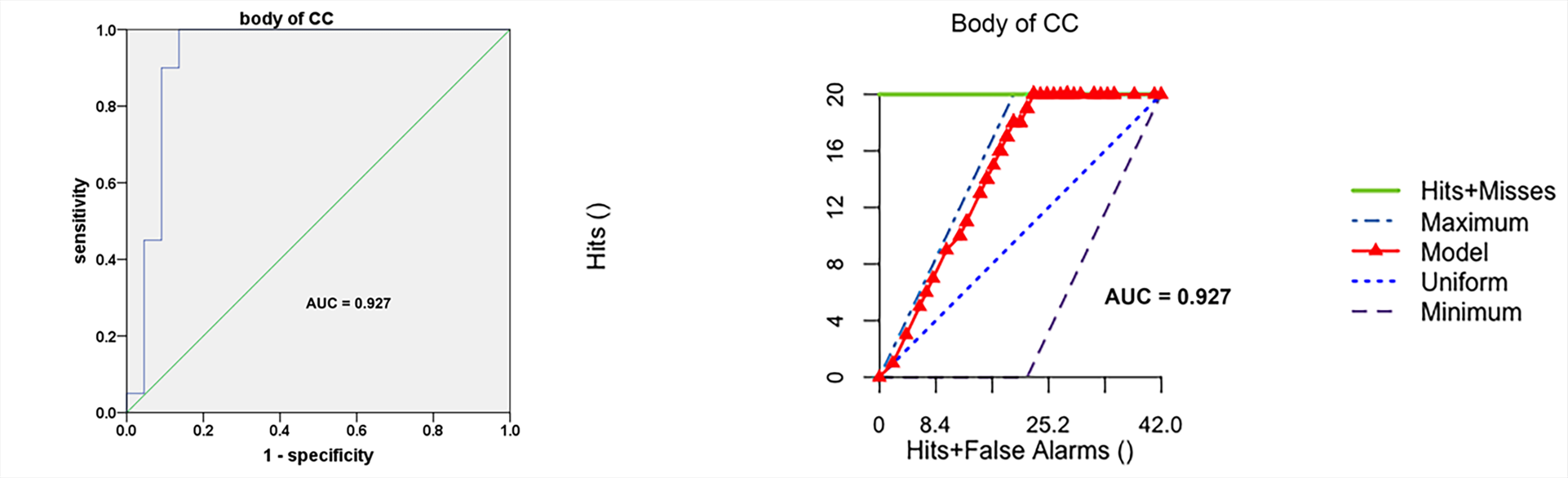

Supplement: Supplementary file 1 [file Image_1.TIF]

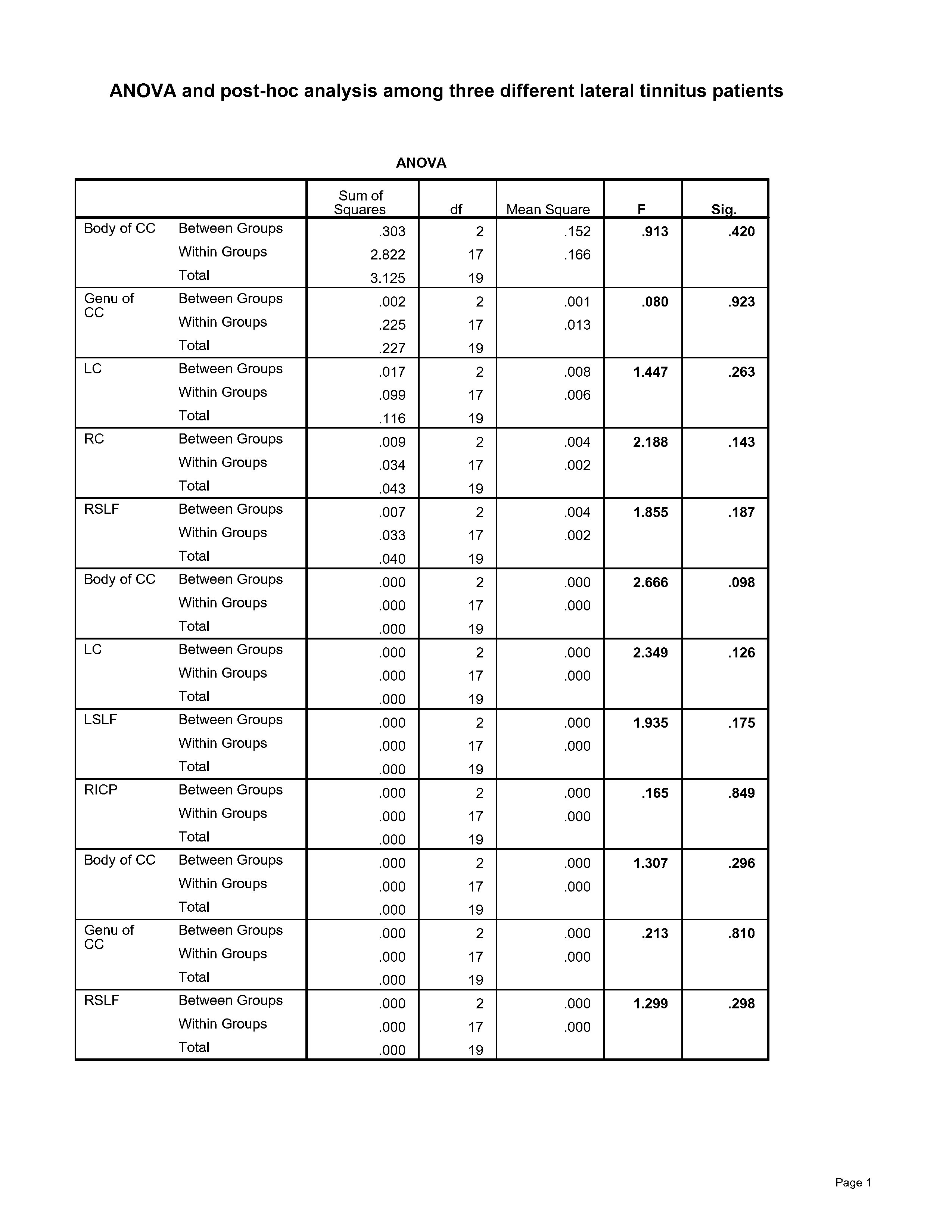

Supplement: Supplementary file 2 [file Image_2.TIF]

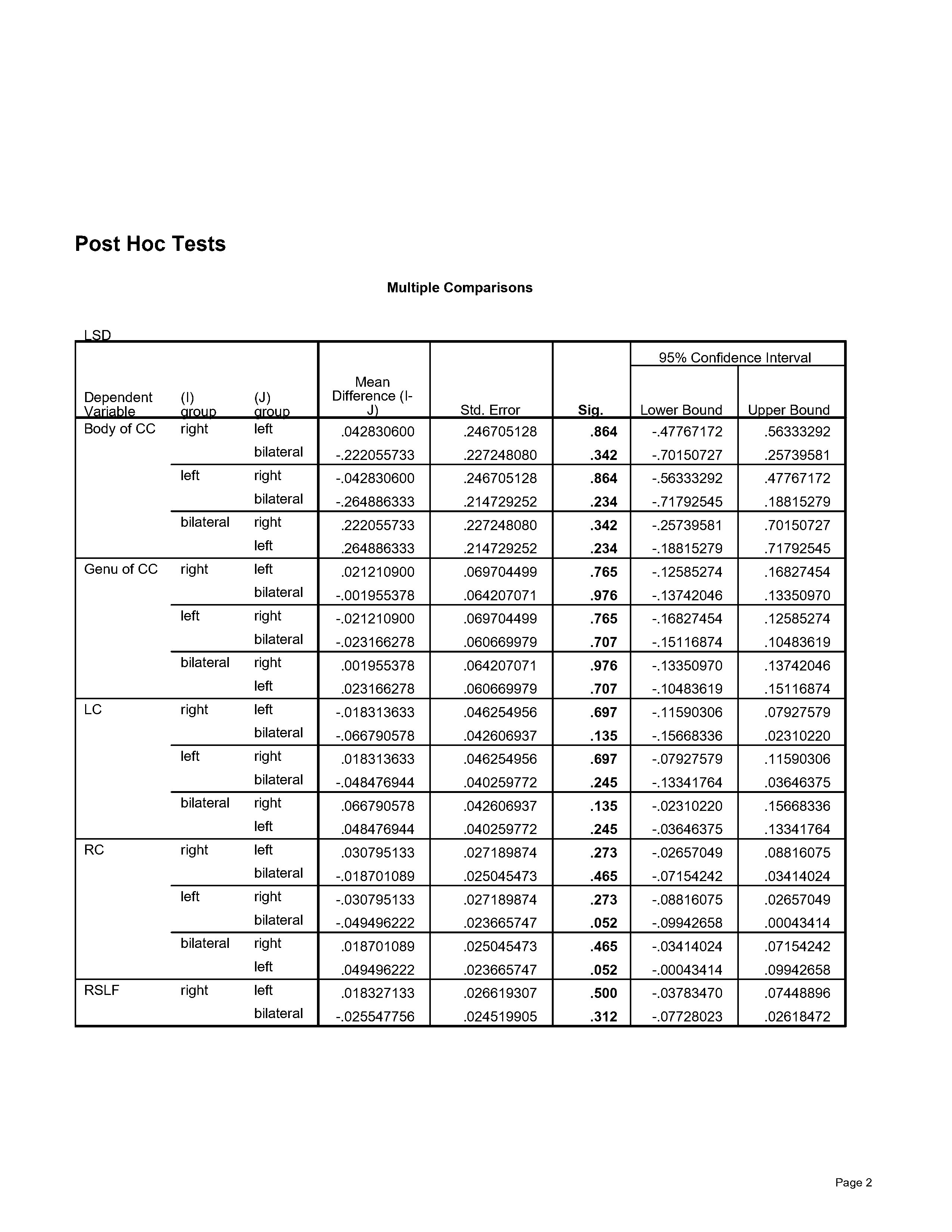

Supplement: Supplementary file 3 [file Image_3.TIF]

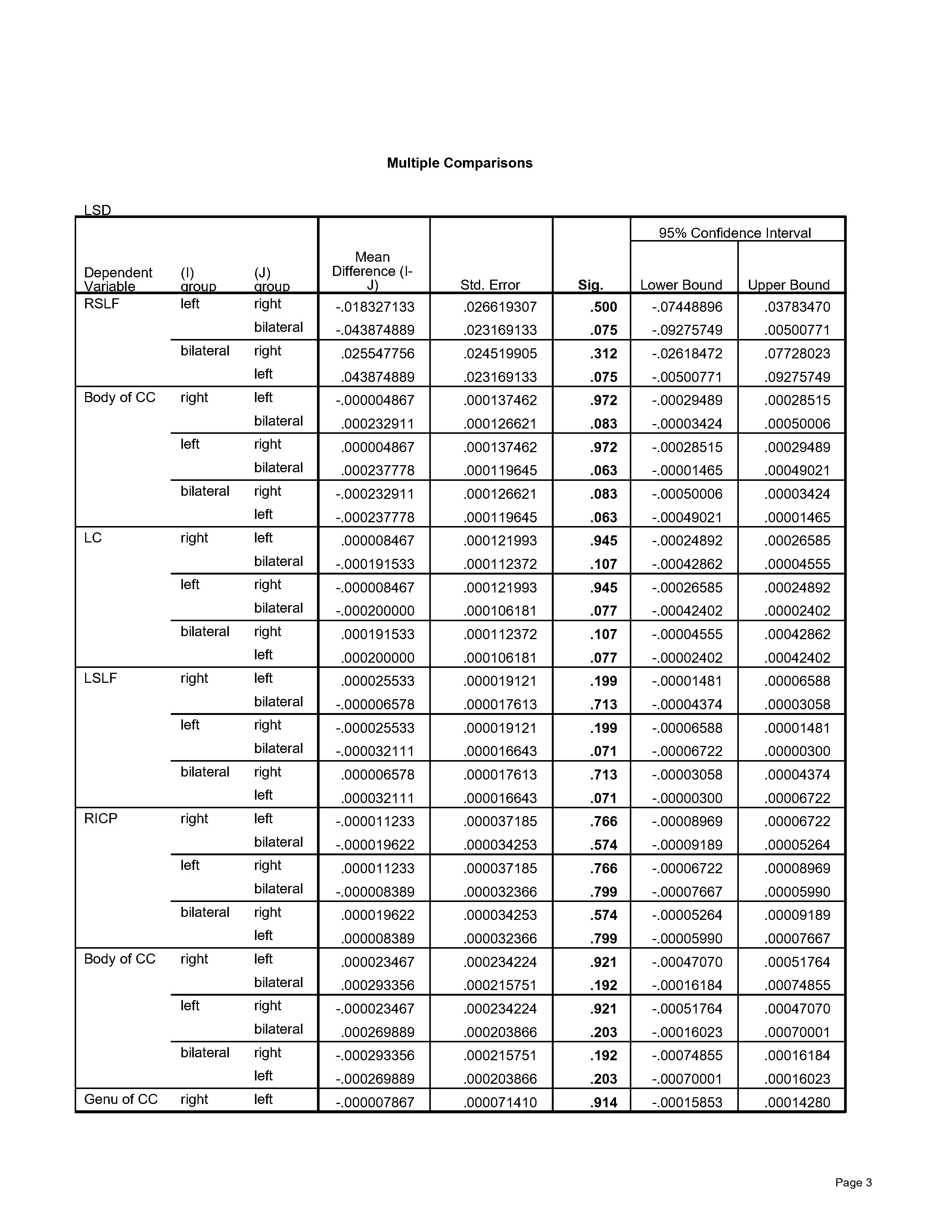

Supplement: Supplementary file 4 [file Image_4.TIF]

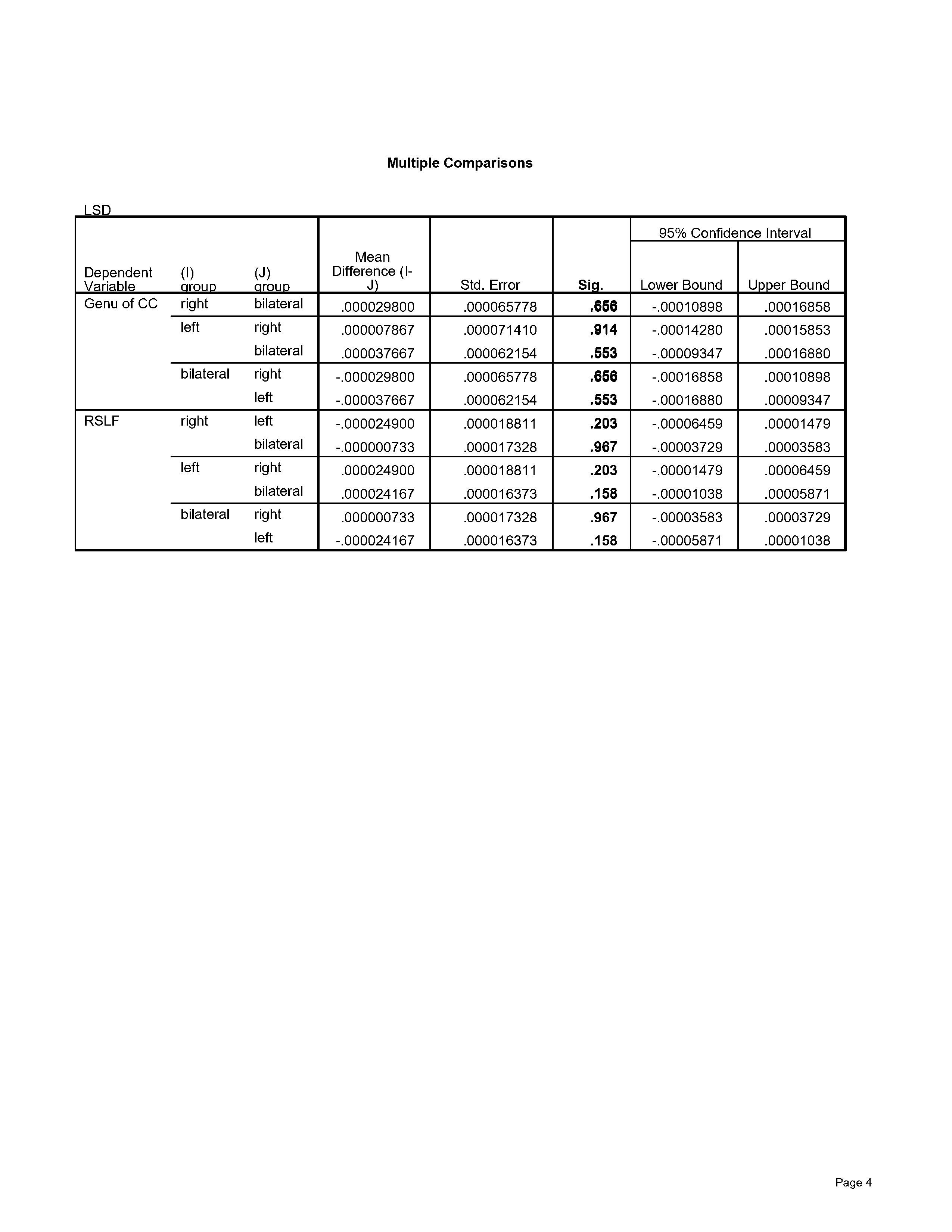

Supplement: Supplementary file 5 [file Image_5.TIF]
